# Vasopressor use in partial flap necrosis in free flap transplant patients with vascular comorbidities: A retrospective study

**DOI:** 10.1016/j.jpra.2024.06.018

**Published:** 2024-07-06

**Authors:** Nadjib Dastagir, Doha Obed, Florian Bucher, Jana L. Schmidt, Khaled Dastagir, Peter M. Vogt

**Affiliations:** Department of Plastic, Aesthetic, Hand and Reconstructive Surgery, Hannover Medical School, 30625, Hannover, Germany

**Keywords:** Reconstructive surgery, Free flap, Microsurgery, Vasopressors

## Abstract

Intraoperative use of vasopressors in free flap surgeries is controversially debated. The predominant concern is that pedicle blood supply will decrease leading to post-operative complications. This study examined the role of intraoperative vasopressors, specifically norepinephrine, in free flap partial necrosis based on the patients’ comorbidities. We retrospectively analyzed 192 patients who received free flap treatment between 2006 and 2021 and were stratified based on vascular comorbidities. We assessed the role of intraoperative vasopressors using multivariate analysis. Patients who were administered vasopressors did not have a significantly higher risk of partial flap necrosis compared to patients who were not administered vasopressors (OR: 1.439, 95% CI: 0.618-3.348, *p*=0.399). Upon stratifying by vascular comorbidities, we found that patients with two or more vascular comorbidities who were administered vasopressors had a significantly higher risk of developing flap necrosis (OR: 3.882, 95% CI: 1.266-14.752, *p*=0.046), indicating that vasopressor use in patients with multiple vascular comorbidities is a risk factor for partial flap necrosis. To minimize the risk of flap marginal necrosis in patients with vascular comorbidities, we recommend limited use of vasopressors or minimizing the flap area to preserve vascularization.

## Graphical abstract

**Figure:**
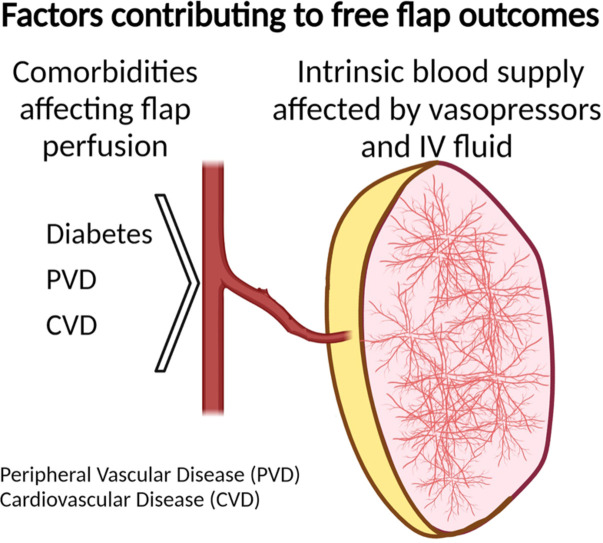

## Introduction

The surgical free flap is a well-established technique used to reconstruct skin and muscle defects. In this technique, a piece of healthy tissue is disconnected from its blood supply, transplanted, and reconnected to the blood supply at the defect site. As a sophisticated microsurgical method, this operation is normally only used for extensive defects such as those occurring after traumatic injury or tumor sectioning.^1^, ^2^, ^3^ A major advantage of these operations is the high success rate, which averages approximately 98%; however, there is a global revision rate of 6% underscoring the importance of identifying factors that predict flap success.^4^^,^^5^ The reliability of this method is due to a combination of refined surgical instruments, the advancement in microscopy, and continuously improving microsurgery techniques and training.^6^^,^^7^ Although the free flap has a history of success, the risk of partial flap necrosis and post-operative complications remains a concern for several surgeons.^8^ The flap perimeter can be particularly susceptible to necrosis and can lead to prolonged hospitalization, increased healthcare costs, and reduced patient satisfaction.^9^, ^10^, ^11^ Therefore, understanding the risk factors associated with free flap complications is important in the field of microsurgery to improve operative success and healing outcomes.

Assessing risk factors during the intraoperative period is a major target in predicting the success of free flaps. By understanding which factors are significantly related to flap failure and post-operative complications, surgeons can mitigate risks.^12^ One factor that has been controversially debated is the intraoperative administration of catecholamines.^13^^,^^14^ Several microsurgeons avoid the use of catecholamines due to the fear of a worse outcome. As vasopressors, catecholamines induce vasoconstriction that might decrease tissue perfusion and thus contribute to flap necrosis. Despite this common assumption, several studies indicate that administration of vasopressors does not impair free flap survival, as previously feared.^13^ A meta-analysis by Goh et al. examined 8,653 cases and found that perioperative administration of vasopressors had no detrimental effects on free flap survival.^13^ Additionally, a single-institution study by Fang et al. found that intraoperative use of the vasopressors, phenylephrine, ephedrine, and calcium chloride, did not increase flap failure rates in patients with cancer.^15^ Although the use of vasopressors did not appear to directly influence flap failure outcomes, it is important to consider patient risk factors that might, in combination with intraoperative vasopressor administration, increase the likelihood of flap complications. One study examined the impact of vascular risk factors on flap failure and found that patients with diabetes mellitus were at a significantly higher risk of complications; however, other vascular comorbidities such as smoking, arterial hypertension, and peripheral arterial vascular disease were not investigated in association with catecholamine use.^16^

Another important consideration in free flap complications is the extent of necrosis. Although free flap transplantation usually has a high degree of success, depending on the extent of necrosis the flap may require salvaging and subsequent surgery.^8^ Partial flap necrosis is an important aspect of the free flap surgery and a healthcare concern owing to the need for extended hospital stays and increased risks of further complications including flap failure. Therefore, it is essential to consider how intraoperative vasopressor use and patient comorbidities interact and assess their effect on flap survival during transplantation. In our study, we examined the relationship between vasopressor use (norepinephrine) and vascular comorbidities in free flap patients to assess their effects on flap necrosis and help microsurgeons identify patient risk factors.

## Materials and methods

### Study design

This study was designed as a retrospective single-center study to evaluate the role of intraoperative vasopressor use on free flap outcomes. The types of free flaps studied included fasciocutaneous and musculocutaneous flaps. Flaps examined in this study occurred on the trunk and extremities, and flaps on the head and neck were not considered. Following flap transplantation, anastomosis was successfully achieved in all patients. Anastomosis success was determined by a warm surface temperature, a capillary refilling time of 3 s, and the detection of intravascular arterial and venous blood flow using an acoustic cutaneous Doppler. Prior to the development of partial flap necrosis, there were no instances of revision surgery among these patients. A total of 192 patients were analyzed in this study. Patients received free flap treatment between 2006 and 2021. Study eligibility included being at least 18 years of age and having a good understanding of the German language. Consent forms were signed and personally dated by the patients and surgeons prior to participation. This study was performed in accordance with the ethical standards of the Declaration of Helsinki and was approved by the local ethical review committee [No. 7887_BO_K_2018].

### Assessment of flap outcomes

Patient history data were collected regarding vascular comorbidities and smoking status. Vascular comorbidities examined in this study were limited to peripheral artery disease (PAD) and diabetes based on the frequency within the patient population. In this study, smoking was also included as a vascular comorbidity due to the resulting endothelial dysfunction that could compromise vascular integrity. Flap necrosis was assessed by imaging the necrotic region using a CANON EOS 4000D and analyzing the total area using FIJI. Blood perfusion in the flap was measured using laser speckle contrast analysis (LASCA). Perfusion units (PU) were calculated using the PIMsoft software analysis tool (Perimed, Sweden).

### Statistical analysis

Statistical significance between two groups was analyzed using univariate analysis, multivariate analysis, and *t*-test. Results are presented as mean ± SEM. Significance was defined as *p*<0.05.

## Results

### Patient comorbidities significantly increased the risk of partial flap necrosis

The mean age of patients receiving vasopressors (norepinephrine) in this study was 61.2 years (range: 20-79 years) and 51.5 years (range: 19-77 years) in patients not receiving vasopressors. In this study, 40% of study participants were women and 60% were men (Table 1). The mean American Society of Anesthesiology classification was comparable between the vasopressor and non-vasopressor groups, with no vasopressor patients having a score of 2.32 compared to 2.63 of the vasopressor group. Other intraoperative parameters were comparable between these two groups, with no significant differences in operative time and mean arterial pressure (Table 2). Patients who were administered vasopressors had higher fluids compared to patients without vasopressors; however, this difference was not significant. We first examined the total patient population and found no significant increase in risk between vasopressor use and partial flap necrosis (OR: 1.265, 95% CI: 0.509-3.144, *p*=0.613). When the patients were stratified based on one vascular comorbidity and vasopressors administration, significant changes were found (Table 3). Upon examining the patients who had two or more vascular comorbidities, we found a significant increase in their risk of partial flap necrosis development (OR: 3.882, 95% CI: 1.266-14.752, *p*=0.046).

**Table 1:** Patient characteristics.

Characteristics	Vasopressors	No vasopressors
Mean age (years)	61.2±15.7	51.5±12.3
Male	79	36
Female Vascular comorbidity Mean ASA* classification	48 82 2.63±0.5	29 38 2.32±0.6

⁎ American society of anesthesiology.

**Table 2:** Perioperative parameters.

	Vasopressors	No vasopressors	*p* value
OR time (h)	6.54±2.6	6.32±2.3	0.37
MAP* (mmHg)	72±6.6	69±4.9	0.87
Fluids (mL)	5685± 1278	4467± 1767	0.44

⁎ Mean arterial pressure.

**Table 3:** Effect of vasopressors in patients with one or more vascular comorbidity on partial flap necrosis.

Comorbidity	Odd Ratio (95% CI)	*p* value
Diabetes	1.37 (1.209-15.44)	0.049
PAD*	1.55 (1.437-19.49)	0.047
Smoking Vascular comorbidity (≥2)	1.77 (1.023-9.090) 3.88 (1.266-14.752)	0.052 0.046

⁎ Peripheral arterial disease.

### Partial flap necrosis is localized to the flap perimeter

**Figure 1:**
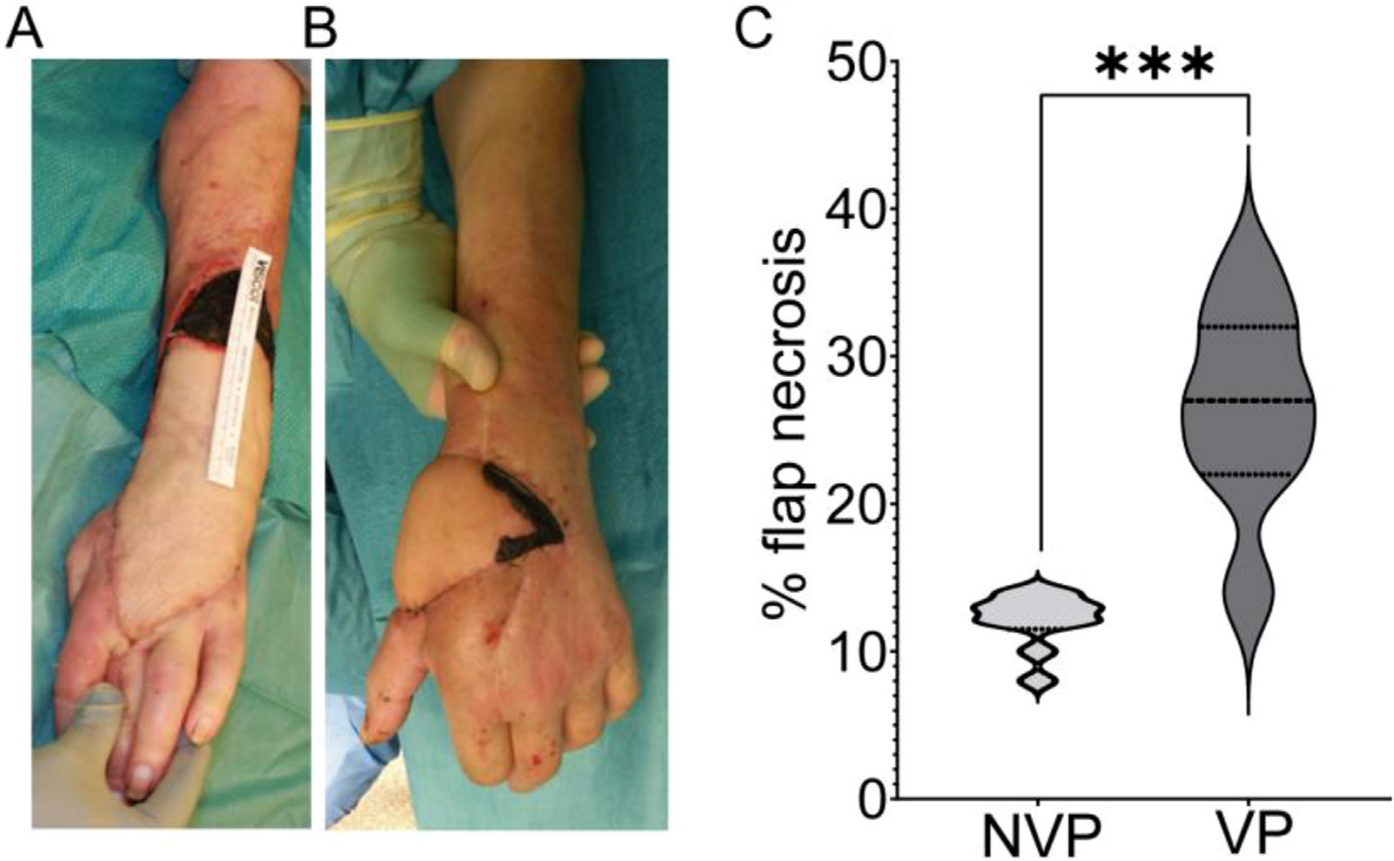
Increased partial flap necrosis in patients with two or more vascular comorbidities following vasopressor use (***p<0.001). (A, B) Representative images of partial flap necrosis developing along the flap perimeter (NVP: no vasopressors; VP: vasopressors). (C) Quantification of percent necrotic area relative to the flap size.

**Figure 2:**
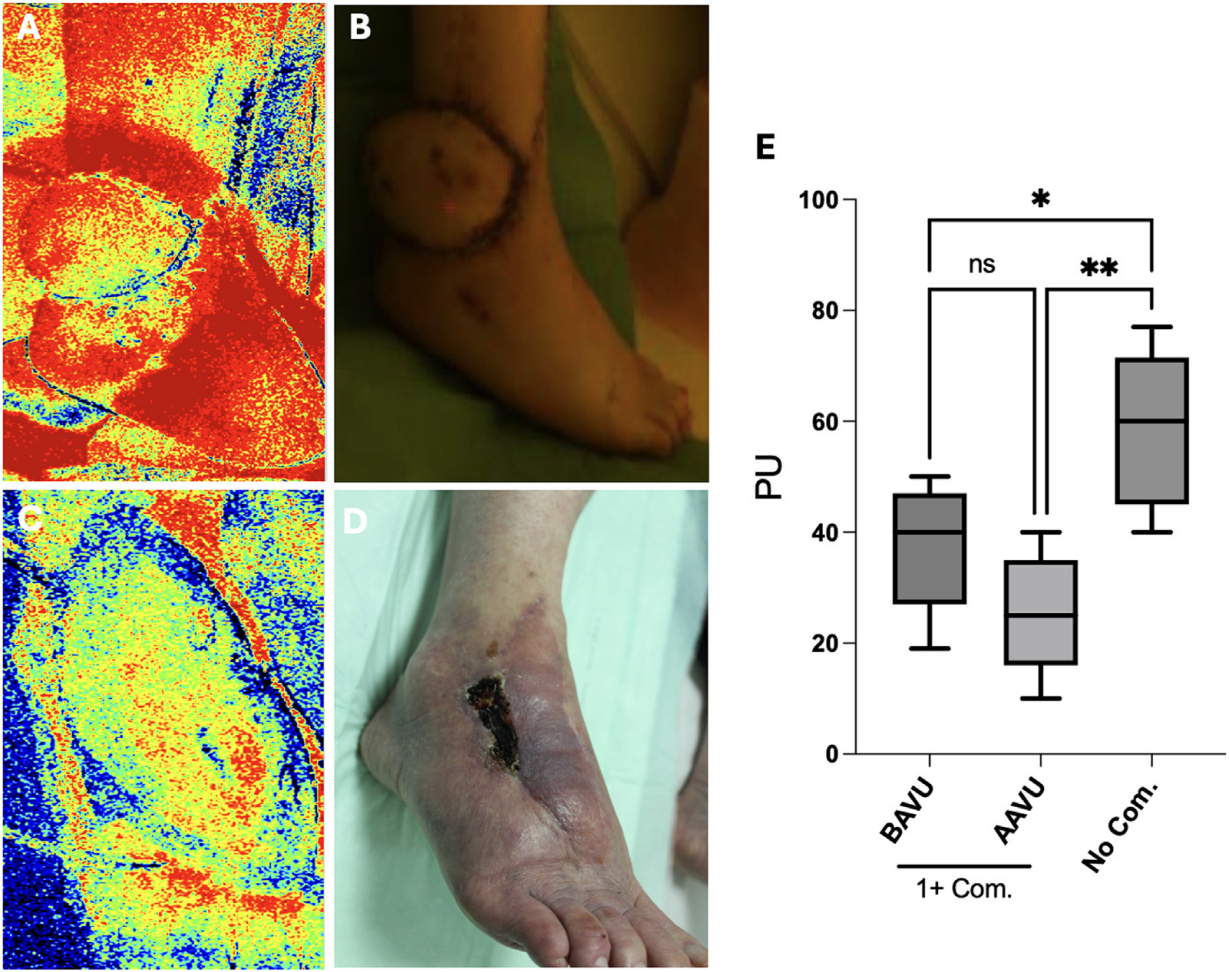
Reduced flap perfusion in patients with vascular comorbidities measured 7 days after transplantation. Representative laser speckle contrast analysis (LASCA) of a patient with no comorbidities (A, B) and a patient with multiple vascular comorbidities (C, D). Quantification of perfusion units in the flap determined using LASCA between patient cohorts (E). (PU: perfusion units; AAVU: above average vasopressor use; BAVU: below average vasopressor use; Com: patients with comorbidities; No Com: patients without comorbidities).

To further understand this relationship between intraoperative vasopressor use and patient comorbidities, we analyzed the localization and extent of necrosis. We found that patients with vascular comorbidities had significantly higher necrotic areas compared to patients without comorbidities who were administered vasopressors. The average necrotic area of patients with comorbidities and vasopressors administration was 28% compared to 9% in patients without vasopressors (Figure 1A-C). Post-operative complications such as partial necrosis result in an increased length of hospital stay. Patients with partial flap necrosis had a longer mean hospital stay of 18 ± 25 days, compared to the average stay of patients without flap necrosis of 11 ± 23 days, leading to increased healthcare costs. In partial flap necrosis cases, the total cumulative intraoperative dose of norepinephrine ranged from 144-941 µg, with an overall mean dose of 382 µg. In patients who did not experience partial flap necrosis, the overall dosage of norepinephrine was 344 (0-757) µg over the course of the surgery, demonstrating that a lower overall norepinephrine dosage was used in cases without flap necrosis compared to cases with necrosis, although this difference was not statistically significant. Patients with at least one comorbidity had significantly reduced flap perfusion, quantified in perfusion units (PU) using LASCA, compared to patients without comorbidities (Figure 2A-E). The reduced perfusion occurred in patients with comorbidities who were treated with above average dose of vasopressors (>382 µg) as well as below average use of vasopressors (<382 µg), with the strongest decrease in perfusion occurring in the former group.

## Discussion

Intraoperative administration of vasopressors during free flap transplantations has been widely debated among microsurgeons for its potential negative effects on flap vascularization due to vascular pedicle vasospasm.^17^, ^18^, ^19^ Notably, vasospasm can also occur in the absence of vasopressor use; however, the mechanisms of vasospasm are complex and may be influenced by vasopressors.^20^ In this study, we found that patients with more than one vascular comorbidity receiving intraoperative vasopressors had an increased odds ratio for developing partial flap necrosis. Partial flap necrosis can lead to further complications such as subsequent reconstructive surgery, prolonged hospital stay, and increased risk of infection.^9^ This relationship between intraoperative vasopressor use and vascular comorbidities highlights the importance of assessing patient risk factors prior to free flap transplantation to modify surgical plans to minimize the risk of necrosis or flap failure.^11^

Microvascular surgeons are often reluctant to use vasopressors during free flap transplantation owing to the possibility of vasoconstriction and blood supply reduction to the flap. A major factor in the success of a free flap transplant is the re-anastomosis of the flap's artery and microvasculature to the local blood supply.^21^ In cases where the vasculature is compromised, re-exploration at the transplant site and revision surgery may be required, which can increase the risk for further complications. One study examining complications after head and neck free flaps found that re-exploration and vascular compromise were significantly associated with Charlson's comorbidity index.^22^ Therefore, it is essential that microsurgeons understand patient risk factors that are associated with free flap vascular complications. Recent meta-analyses and reviews corroborate our finding that vasopressor administration alone does not significantly increase the risk of partial flap necrosis when comparing patients who were administered intraoperative vasopressors to those who were not.^23^^,^^24^ Although vasopressor administration alone did not significantly increase the risk of partial flap necrosis in the overall study population, the presence of pre-existing vascular comorbidities emerged as a crucial risk factor. Patients with multiple vascular comorbidities such as PAD, diabetes mellitus, and smoking history exhibited a substantially higher likelihood of developing partial necrosis when exposed to intraoperative vasopressors (Table 3).

Our study found that patients with two or more vascular comorbidities who were administered vasopressors had a significantly higher risk of developing partial flap necrosis compared to those without comorbidities (OR: 3.882, 95% CI: 1.266-14.752, p=0.046). Patients with vascular comorbidities and vasopressor use also had significantly larger necrotic areas (28%) compared to those without comorbidities (9%) (Figure 1). Furthermore, patients with at least one comorbidity had significantly reduced flap perfusion, measured using LASCA, compared to patients without comorbidities (Figure 2), demonstrating that reduced circulation in these patients corresponds to the development of necrosis.

Low-dose norepinephrine infusions were predominantly used and administered as a continuous low-dose infusion in the range of 0.02-0.05 µl/kg/min rather than as a bolus dose. In cases without partial flap necrosis, a lower overall norepinephrine dosage of 344 (0-757) µg was used compared to 382 (144-941) µg in cases with necrosis; however, the difference in dosage was not significant. This suggests that although higher cumulative norepinephrine doses may have been used in cases that developed partial necrosis, the dosing was still within the low-dose infusion range aimed at maintaining adequate perfusion without compromising the flap in both groups.

Vasopressor use and low mean arterial pressure (MAP) can potentially compromise flap perfusion. Previous literature suggests that cumulative intraoperative hypotension, defined as MAP below 60 mmHg, could be a predictor of unfavorable post-operative outcomes.^36^ Burkhard et al. found that cumulative hypotension did not increase the risk of flap failure or systemic complications in patients with fibula free flap and neck reconstructions.^37^ However, the prudent use of vasopressors to maintain an adequate perfusion pressure may be preferable to allowing severe hypotension. Further work investigating the importance of mean arterial pressure compared to vasopressor use would better inform clinicians on the role of these factors on flap survival and post-operative complications.

The investigation of the impact of intraoperative vasopressor administration on flap outcomes is a crucial area of research, as it provides valuable insights for microsurgeons in their decision-making process regarding the use of vasopressors by considering the specific characteristics of each patient. We theorize that the reason for the increased risk of flap necrosis in patients with vascular comorbidities is due to pre-damaged choke vessels in the flap. Choke vessels comprise the veins and arteries traveling between two angiosomes.^25^ Angiosomes are defined as three-dimensional tissue regions supplied by a specific arterial and venous source.^26^ The preparation for a free flap transplant requires the isolation of a flap on one vascular pedicle and reattaining adequate blood flow resistance in these choke vessels to allow them to be fully opened.^27^^,^^28^ When the choke vessels become damaged, such as from a vascular comorbidity, it compromises the vascularization of the flap and the resulting decreased blood perfusion can lead to necrosis. Moreover, denervation of the vessels might play an important role in vasodilatation and formation of choke vessels.^29^ We observed that patients with one or more comorbidities had significantly reduced perfusion units in the flap compared to patients without comorbidities (Figure 2), supporting the hypothesis that damaged vessels in patients with comorbidities contributes to reduced flap perfusion and flap necrosis. We believe the choke vessels specifically are affected based on the localization of the necrosis. The distal perimeter of the flap was particularly susceptible to necrosis in the patients with vascular comorbidities, with a similar location to that of the choke vessels.

Another potential cause for the partial flap necrosis observed in patients with vascular comorbidities is the ischemia reperfusion response. During a free flap transfer, the donor site must undergo ischemia followed by microanastomosis and restoration of blood perfusion at the transplant site. One study that examined the effect of this ischemia and reperfusion in human muscle free flaps found significant upregulation of inflammation and angiogenesis markers coupled with interstitial edema.^30^ This inflammatory environment has been shown to play an important role in choke vessel remodeling and arteriogenesis.^31^^,^^32^ Several vascular comorbidities are associated with changes in the immune system which can negatively affect wound healing. For example, wound healing studies in patients with diabetes have found that the dysfunction of the immune cells, particularly macrophages, neutrophils, and T cells, involved in regulating inflammation can contribute to the delayed healing outcomes.^33^ Furthermore, smoking is known to cause endothelial dysfunction from nitric oxide bioavailability, which can exacerbate vascular problems.^34^ When a vascular comorbidity generates changes in the immune and endothelial cell environment, it could lead to an abnormal healing response and complications such as the necrosis observed in this study. Further characterization of the free flap immune cell populations would be an interesting direction for future work to better elucidate the mechanisms regulating free flap vascularization and healing.

Flap size also plays an important role in flap outcomes and healing. The larger the flap, the higher its vascular needs; therefore, facilitating proper blood perfusion is an important consideration in free flap transplantation. One study examined the risk factors associated with pedicle flap necrosis in hand reconstruction and found that flap size is the second most significant risk factor.^35^ For patients with multiple vascular comorbidities, we recommend limiting the flap size to prevent further perfusion complications and decrease the risk of partial flap necrosis.

Limitations of this study include the restriction on the number of vascular comorbidities examined. As a single-institution study, the diversity of the patient population was limited and we chose to restrict the vascular comorbidities examined to enable adequate sample sizes for analysis. Future analysis with a wider range of patients such as a multi-institution analysis would be useful to understand the relationship between vascular comorbidities, intraoperative vasopressors, and free flap outcomes. Another important limitation in vasopressor studies is the clinical need to keep the mean arterial pressure >65 mmHg for proper organ perfusion. Patients with vascular comorbidities, such as PAD, are more likely to be administered vasopressors during an operation to maintain the minimum pressure. This leads to more patients with vascular comorbidities receiving vasopressors compared to the patient population without these comorbidities.

## Conclusions

Patients with one or more vascular comorbidities are at a higher risk for partial flap necrosis following vasopressor administration compared to patients without these comorbidities. Partial flap necrosis can prolong hospital stays and might require subsequent surgery depending on the extent of tissue damage. If a patient with multiple vascular comorbidities requires a fasciocutaneous free flap, we recommend avoiding or limiting the use of intraoperative vasopressors to reduce the risk of partial necrosis. We also recommend minimizing the area of the free flap in such patients to prevent inadequate flap perfusion.

## Funding

No funding.

## Institutional review board statement

The study was approved by the local ethical review committee [No. 7887_BO_K_2018].

## Informed consent statement

Informed consent was obtained from all subjects involved in the study.

## CRediT authorship contribution statement

**Nadjib Dastagir:** Data curation, Formal analysis, Methodology, Visualization, Writing – original draft, Writing – review & editing. **Doha Obed:** Methodology, Writing – review & editing. **Florian Bucher:** Formal analysis, Writing – review & editing. **Jana L. Schmidt:** Formal analysis, Writing – review & editing. **Khaled Dastagir:** Conceptualization, Supervision. **Peter M. Vogt:** Conceptualization, Supervision, Writing – review & editing.

## Declaration of competing interest

The authors declare no conflict of interest.
